# Robotic Transthoracic Repair of a Right-Sided Traumatic Diaphragmatic Rupture

**DOI:** 10.1055/s-0040-1716330

**Published:** 2020-09-28

**Authors:** Jin K. Kim, Anand Desai, Anastasia Kunac, Aziz M. Merchant, Constantinos Lovoulos

**Affiliations:** 1 Department of Surgery, Rutgers New Jersey Medical School, Newark, New Jersey; 2 Division of Trauma and Critical Care, Rutgers New Jersey Medical School, Newark, New Jersey; 3 Division of General and Minimally Invasive Surgery, Rutgers New Jersey Medical School, Newark, New Jersey; 4 Division of Cardiothoracic Surgery, Rutgers New Jersey Medical School, Newark, New Jersey

**Keywords:** traumatic diaphragm rupture, laparotomy, minimally invasive surgery, robotic surgery

## Abstract

**Introduction** Traumatic diaphragm rupture injury repairs are predominately performed through thoracotomy, laparotomy, or a combination of the two approaches. While open surgery is often necessary to follow the fundamentals of damage-control operations in unstable or polytrauma patients, minimally invasive surgery may be an alternative for those with a low injury burden to reduce the postoperative morbidities associated with open operations.

**Case Description** We describe the first case of a right-sided diaphragm rupture from blunt trauma that was repaired by a robotic transthoracic approach in the index admission.

**Conclusion** Minimally invasive repair of an acute traumatic diaphragm rupture is feasible in selected trauma patients.

Traumatic diaphragm rupture from a blunt injury is uncommon but is associated with a 28% mortality risk and significant morbidity. 1 The diagnosis of traumatic diaphragm rupture is often delayed due to the occult nature of associated symptoms and signs. 2 3 Computed tomography (CT) may allow an earlier diagnosis in stable patients. 3 Left-sided diaphragm ruptures are more common, but right-sided injuries result in a higher mortality risk. 1 4 In an acute setting, the morbidity and mortality of traumatic diaphragm rupture are associated with concurrent thoracoabdominal injuries, while in a delayed setting, are associated with intestinal herniation and subsequent strangulation. 4 Surgical approaches through laparotomy, thoracotomy, or thoracoabdominal incisions are often offered to repair traumatic diaphragm ruptures; however, these open operations are associated with a 20% pulmonary complication rate postoperatively. 1 5

While it is prudent to perform open surgery in unstable patients or in patients with associated organ injuries that require urgent repair, minimally invasive surgery (MIS) in a delayed setting is feasible in selected patients. 1 4 5 6 7 As MIS has been shown to reduce respiratory complications, pain, and faster recovery compared with open surgery, incorporating MIS in selected trauma patients may spare them from the morbidities of open operations. 8 Several centers have published case reports of thoracoscopic or laparoscopic repair of traumatic diaphragm ruptures in delayed settings with either primary repair with nonabsorbable sutures or patch closure of the diaphragm with mesh. 6 Here, we report the first case of a robotic transthoracic repair of a right-sided traumatic diaphragm rupture in an acute setting during the index admission.

## Case Presentation

**Fig. 1:**
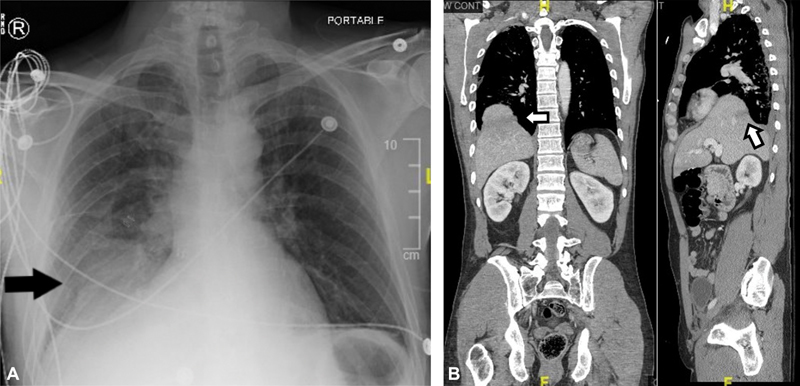
( **A** ) *Preoperative chest radiograph* . Chest radiograph taken during the trauma survey. Black arrow points to right-sided haziness in the chest. There is no evidence of rib fractures. ( **B** ) *Preoperative CT* . CT of the chest, abdomen, and pelvis taken after primary and secondary survey. Mild pleural effusion, likely representing hemothorax, is present at the right costophrenic angle. White arrow on the left points to the collar-sign of liver suggestive of diaphragmatic herniation. White arrow on the right points to the grade 2 hepatic laceration. There were no other significant thoracoabdominal organ injuries. CT, computed tomography.

A 45-year-old male with a history of chronic obstructive pulmonary disease presented as a restrained driver in a low speed motor vehicle collision. His chief complaint was left wrist and hip pain. He denied shortness of breath, hematemesis, or chest pain. He was hemodynamically stable with unremarkable findings on primary survey. On secondary survey, he had tenderness of the left wrist and hip. His chest radiograph was notable for haziness above the right hemidiaphragm ( Fig. 1A ). CT of the chest, abdomen, and pelvis revealed a collar sign on the dome of the liver, suggestive of a right diaphragmatic rupture, as well as a grade 2 liver laceration on segment VIII, L1 and L2 transversus process fractures, an inferior pubic rami fracture, and a right acetabular fracture ( Fig. 1B ). Additional radiographs also revealed a left wrist fracture.

Emergent open repair of the traumatic diaphragm rupture was deferred in this patient since it carried a substantial risk of postoperative respiratory failure. 9 Instead, we chose to closely monitor him after the repair of his orthopaedic injuries. As he remained hemodynamically stable with good respiratory capacity over 48 hours, we felt that he was a candidate for minimally invasive repair of his diaphragm injury. We offered a robotic transthoracic repair to avoid the complications associated with an open approach.

## Description of Procedure

**Fig. 2:**
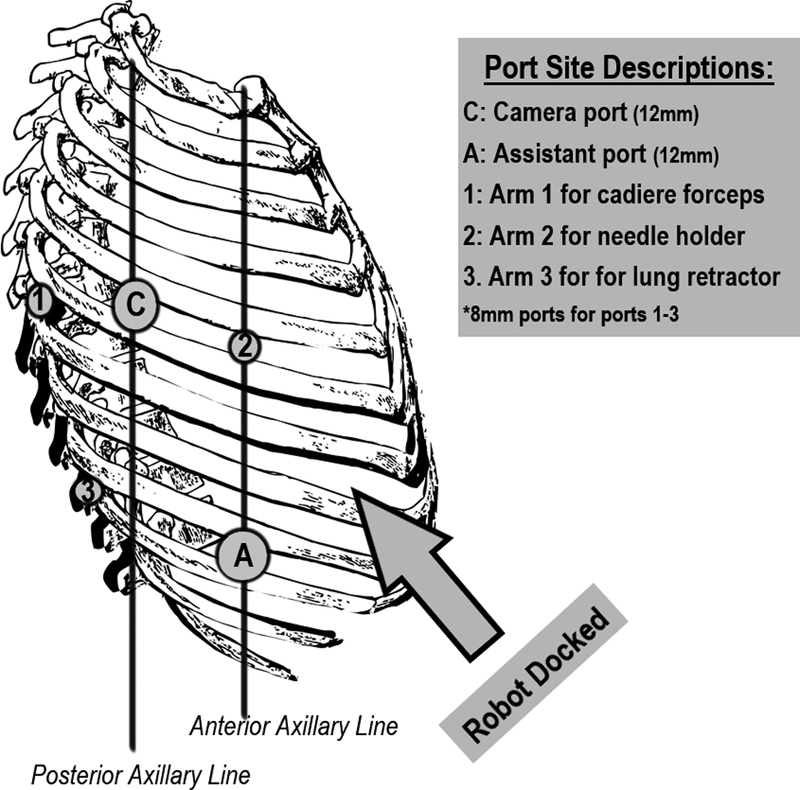
*Trocar placement diagram* . A total of five trocars were placed with the patient positioned in left lateral decubitus. Trocar C: camera port (12 mm). Trocar A: assistant port (12 mm). Trocars 1–3 for the robotic arms (8 mm). Refer to the description of procedure for the details of trocar placement sites.

After induction of general anesthesia via a double lumen endotracheal tube, the patient was placed in the left lateral decubitus position. After bed flexion, the patient was placed in slight reverse Trendelenburg. Single left lung ventilation was initiated. A 12-mm camera port (Trocar C, Fig. 2 ) was inserted at the fifth intercostal space at the posterior axillary line. Under camera visualization, the other three 8-mm trocars (Trocars 1, 2, and 3; Fig. 2 ) were inserted and CO2 was insufflated. Trocar 1, which was used for arm 1 of the robot, was placed was placed 10 cm posterior to Trocar C. This arm was equipped with cadiere forceps. Trocar 2 was placed 10 cm anteriorly to Trocar C and was used to hold the needle for suturing later in the case. Trocar 3 was placed two fingerbreadths lateral to the spinal processes at the nineth intercostal space. This port was used mainly to retract the lung robotically. A 12-mm assistant port (Trocar A) was inserted approximately at the anterior axillary line at the nineth intercostal space. *Da Vinci Si* robot was brought in from the patient's left side cephalad from the legs at a 30-degree angulation and docked. The patient was facing the mainframe of the robot. The rationale for positioning the ports this way was because this patient's diaphragm injury extended medially toward the retrocardiac region so we thought docking the robot from the patient's left side would allow easier access to the injury. Placing the assistant port anteriorly limited the assistant's working space due to the robot, but we wanted to avoid placing this port posteriorly as this approach would require us to cut through more muscle (the trapezius and rhomboids) and in theory, increase the morbidity of the operation.

We first started with inspection to assess the extent of the injury. A significant amount of blood clot was suctioned, but we did not see any active bleeding. The dome of the liver was herniating into the right chest. Through Trocar 3, the lung retractor was placed and the right lung was retracted superiorly for better exposure. The liver was gently reduced back to the abdomen with robotic cardieres. The defect in the diaphragm measured approximately 9 × 11 cm. The wound edges appeared healthy so we did not resect or debride any part of the diaphragm. The diaphragm was then repaired primarily with 2–0 Ethibond pledgeted sutures in an interrupted U-stitch fashion. The medial posterior portion of the rupture near the pericardial reflection was reapproximated in a figure-of-eight fashion. Excess diaphragmatic tissue over the first layer of closure was then reapproximated with a running V-Loc size 0 suture. After undocking the robot and removing all the trocars, one 28-French chest tube was placed through the incision for Trocar A. The patient was repositioned to supine and needle decompression of the intra-abdominal air was then performed at the right upper quadrant.

Our patient was extubated on postoperative day 1 and the chest tube was removed on postoperative day 4. He was clinically stable for discharge by postoperative day 7. He has been followed in clinic for 6 months with good respiratory function and a normal chest X-ray.

## Discussion

Despite the known benefits of MIS compared with open surgery, such as sparing of major muscles, decreased postoperative pain, and respiratory complications, 7 8 10 there is a paucity of literature describing its use in a trauma setting. MIS can be feasible and effective for patients with a traumatic diaphragm rupture with no significant thoracoabdominal organ injuries. 2 6 7 10 In our patient's case, the only thoracoabdominal injury besides the diaphragm rupture was a grade 2 liver laceration, which is often managed nonoperatively. To rule out any major-associated injuries that could have been missed from the initial work-up, we closely monitored our patient for 48 hours. As he remained stable during this time, we felt that he was a candidate for minimally invasive surgery. We decided on a thoracic approach as laparoscopic repairs sometimes require the mobilization of the liver in right-sided traumatic diaphragm injuries. 2

To our knowledge, our experience is the first report on a robotic repair of a right-sided traumatic diaphragm rupture during the index admission. To perform MIS in a trauma setting, careful patient assessment and selection is crucial. 8 Early repair of traumatic diaphragm injuries can be technically easier, as pleuro-visceral adhesions have not yet formed, 6 and prevent complications including bowel obstruction and strangulation that arise when postponing repair. 2 The robotic approach allowed better visualization at the cardio-phrenic and costophrenic reflections. It also allowed better ergonomics and easier suture mobility to reapproximate the diaphragm versus video-assisted thoracoscopy.
